# Genome-wide survey and genetic characteristics of *Ophichthus evermanni* based on Illumina sequencing platform

**DOI:** 10.1042/BSR20220460

**Published:** 2022-05-27

**Authors:** Tianyan Yang, Zijun Ning, Yuping Liu, Shufei Zhang, Tianxiang Gao

**Affiliations:** 1 Fishery College, Zhejiang Ocean University, Zhoushan 316022, China; 2 Guangdong Provincial Key Laboratory of Fishery Ecology and Environment, South China Sea Fisheries Research Institute, Chinese Academy of Fishery Sciences, Guangzhou 510300, China

**Keywords:** gene annotation, genome survey sequencing, microsatellite loci, Ophichthus evermanni, phylogenetic relationship

## Abstract

Ophichthidae fishes limit to continental shelf of all tropical and subtropical oceans and contain more than 350 species, representing the greatest specialization diversity in the order Anguiliformes. In the present study, we conducted a genome survey sequencing (GSS) analysis of *Ophichthus evermanni* by Illumina sequencing platform to briefly reveal its genomic characteristics and phylogenetic relationship. The first *de novo* assembled 1.97 Gb draft genome of *O. evermanni* was predicted based on K-mer analysis without obvious nucleotide bias. The heterozygosity ratio was 0.70%, and the sequence repeat ratio was calculated to be 43.30%. A total of 9016 putative coding genes were successfully predicted, in which 3587 unigenes were identified by gene ontology (GO) analysis and 4375 unigenes were classified into cluster of orthologous groups for enkaryotic complete genomes (KOG) functional categories. About 2,812,813 microsatellite motifs including mono-, di-, tri-, tetra-, penta- and hexanucleotide motifs were identified, with an occurrence frequency of 23.32%. The most abundant type was dinucleotide repeat motifs, accounting for 49.19% of the total repeat types. The mitochondrial genome, as a byproduct of GSS, was assembled to investigate the evolutionary relationships between *O. evermanni* and its relatives. Bayesian inference (BI) phylogenetic tree inferring from concatenated 12 protein-coding genes (PCGs) showed complicated relationships among Ophichthidae species, indicating a polyphyletic origin of the family. The results would achieve more thorough genetic information of snake eels and provide a theoretical basis and reference for further genome-wide analysis of *O. evermanni.*

## Introduction

Ophichthidae is the family with the most various species in the order of Anguilliformes, which hitherto contains more than 350 valid species belonging to 62 genera all over the world [1]. These snake-shaped fishes are widely spread in tropical and subtropical inshore waters and prefer to slither in muddy substrates or coral reefs by pointed rayless tail tips or acute snouts [2]. Because of less distinguishable morphological features and various shapes of body in different growth stages, it brings great difficulties to effective species identification and phylogeny analysis of this group. The studies on ophichthid eels are limited to morphological identification and new species description [3–8]. There have been no reports on the genome of snake eels until now. The lacking of molecular genetic data has seriously restricted the further evolutionary and genomic studies of Ophichthidae fishes.

Nowadays, high-throughput next-generation sequencing (NGS) provides a more convenient approach to obtain massive genomic sequences, which can more comprehensively reveal the genetic background and phylogenetic relationships at DNA level [9]. With the accomplishment of the first fish whole genome sequencing (WGS) early in 2002 [10], more and more fish genomes have been published, ranging from the model fishes [11,12] to many commercial species [13–17]. The genome survey sequencing (GSS) is a convenient approach to provide fundamental information of genome. It could not only productively identify genome-wide simple sequence repeats (SSRs) but also efficiently predict putative gene functions and targeted the potential exon-intron boundaries [18].

In the present study, we selected *Ophichthus evermanni* [19], a kind of snake eel that mainly distributes in the East China Sea, the South China Sea and the coastal waters of southern Japan as a representative [20], and preliminarily revealed the genomic characterization such as genome size, GC content, heterozygosity and repeat ratio of this snake eel based on genome survey sequencing. Meanwhile, the genome annotation, microsatellite markers identification and mitochondrial genome assembly were conducted by a series of bioinformatics analyses. The information above could be helpful in species identification, adaptive evolutionary mechanisms and phylogenetic studies. Besides, these findings would also supplement the molecular biology data of *O. evermanni* and make a valuable contribution to the genome-wide studies on snake eels.

## Materials and methods

### Sample collecting and DNA extraction

One female specimen of Evermann’s snake eel with body length 771.42 mm and body weight 571.63 g was obtained from coastal waters of Xiamen (118°34′E, 24°15′N), China in December 2020 (Supplementary Figure S1). After identifying it by morphological characteristics and DNA barcoding (mitochondrial DNA COI gene), the examined individual was preserved in −80°C ultra-low temperature freezer, and all animal experiments took place at Fisheries Ecology and Biodiversity Laboratory (FEBL) of Zhejiang Ocean University, Zhoushan, China. Experiments were conducted under the guideline and approval of the Ethics Committee for Animal Experimentation of Zhejiang Ocean University (ZJOU-ECAE20211876).

After species identification and morphological measurement, a piece of fresh muscle tissue was clipped from the base of dorsal fin and soaked in absolute ethanol. The genomic DNA was extracted by using the standard phenol-chloroform method followed by proteinase K digestion to ensure complete protein removal. The DNA integrity was first assessed by 1% agarose gel electrophoresis (5 V/cm, 20 min). And then, the quantity and purity of genomic DNA were checked by Qubit 2.0 fluorometer (Invitrogen, California, U.S.A.) and NanoDrop2000 spectrophotometer (Thermo Fisher Scientific, Delaware, U.S.A.), respectively.

### Library construction and genome survey sequencing

The DNA sample was randomly fragmented into 300–500 bp using Covaris M220 Focused-ultrasonicator (Covaris, Massachusetts, U.S.A.) to construct the two paired-ends sequencing libraries, and then followed by terminal repair, adding an A base to the blunt ends and ligation to sequencing adaptors. After DNA purification and bridge PCR amplification, the prepared DNA library was sequenced based on the Illumina Hiseq 2500 platform with a read length of 2×150 bp by Origin-gene Biomedical Technology Co., Ltd., Shanghai, China (http://www.origin-gene.com/). All sequencing data were deposited in the short-read archive (SRA) database (http://www.ncbi.nlm.nih.gov/sra/) under the accession number PRJNA807805.

### Genome assembly and K-mer analysis

The clean data were obtained after removing reads containing adapters, duplicated reads and low quality reads from the raw genome survey sequence data. All the high-quality reads were assembled based on de Bruijn graph algorithm using SOAP de novo v2.04 software (https://soap.genomics.org.cn/) [21]. Jellyfish software [22] was conducted to count K-mer depth distribution of sequenced reads and then evaluated the genome size according to the formulas: Genome Size = K-mer number/average K-mer depth, Revised Genome Size = Genome Size × (1 − Error Rate). Because the distribution of K-mer frequency yields to Poisson distribution, the peak of K-mer distribution curve can be regarded as the expected depth of K-mer [23]. The heterozygous frequency of the genome of *O. evermanni* was roughly determined based on the K-mer analysis following the description of Liu et al [24]. And the repeat ratio was calculated according to the percentage of the total number of K-mer after the main peak 1.8 times of all K-mer numbers [24,25]. Moreover, the GC content was also an important parameter for measuring the sequencing bias of a genome, which was calculated by the 10 kb non-overlapping sliding windows along the assembled sequence.

### Gene prediction and functional annotation

The software GeneMark-ES (http://exon.gatech.edu/genemark/gmes_instructions.html) [26] was conducted to predict genes. The translated protein sequences were compared with Nr (Non-Redundant Protein Sequence), KOG (Cluster of Orthologous Groups for Enkaryotic Complete Genomes), KEGG (Kyoto Encyclopedia of Genes and Genomes) and GO (Gene Ontology) databases using Blast 2.2.28 + [27], respectively, so as to obtain the annotation information of the predicted genes.

### Microsatellites identification and phylogenetic analysis

The Perl script MicroSatellite (MISA) was used to identify microsatellites in the genome of *O. evermanni* [28]. The settings implemented to detect the minimum numbers of SSRs for mono-, di-, tri-, tetra-, penta- and hexa-nucleotide repeats were as follows: number of mono-nucleotide repeats was less than 10, number of di-nucleotide repeats was less than 6, and numbers of remaining repeats were all less than 5, respectively.

To further reveal the phylogeny of *O. evermanni*, we assembled and generated the complete mitochondrial genome by running a Perl script NOVOPlasty 4.3.1, a *de novo* assembler for organelle genomes from the whole genome data [29]. The circular mitogenome was annotated by the online tool MitoFish (http://mitofish.aori.u-tokyo.ac.jp/) and then checked and corrected the annotation results manually. The complete mitochondrial sequence of *O. evermanni* was submitted to NCBI (National Center for Biotechnology Information) database with the accession number OM421636. The nucleotide composition was calculated by Mega 11 [30]. Twenty Anguilliformes mitogenomes were downloaded from the GenBank, with *Gymnothorax formosus* (GenBank accession number: KP874184) selected as the outgroup. Twelve protein-coding genes (PCGs) excluding ND6 were concatenated for phylogenetic analysis based on Bayesian inference (BI) method inferring by MrBayes 3.2.6 [31] Four independent Markov Chain Monte Carlo (MCMC) chains (one cold chain and three heated chains) were run for 1,000,000 generations with sampling every thousand generations, and then the initial 25% of these sampled trees were discarded as burn in. And before that, assessing nucleotide substitution saturation and selecting the best-fit model of nucleotide substitution were carried out with DAMBE 5.0 [32] and Modeltest 3.7 [33], respectively.

## Results

### Illumina Sequencing data statistics

**Figure 1:**
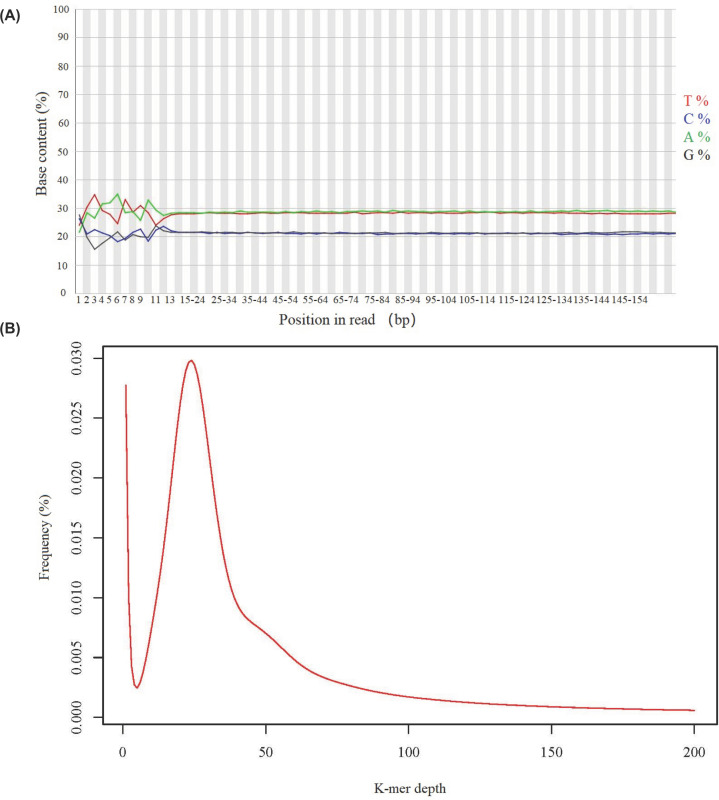
Sequence content across all bases and K-mer (*K* = 17) analysis for estimation of the genome size of *O. evermanni* (**A**) The *X*-axis was the position in read and *Y*-axis was base content. (**B**) The *X*-axis represented K-mer depth and the *Y*-axis was the frequency at a given depth divided by the total frequency of all depths.

The average sequencing depth of the HiSeq data was 50× coverage, which yielded approximately 54.145 Gb clean bases with the error ratio 0.0282% after sequencing quality control. The values of Q20 (base quality > 20) and Q30 (base quality > 30) were 96.575% and 91.525%, respectively, which suggested that the sequencing depth and was sufficient to capture most of the genomic information. The proportions of single base were presented in Figure 1A, the GC content was 42.66% with no apparent abnormalities and obvious GC bias being observed. Ten thousand pairs of reads data were randomly selected from the filtered high-quality data, and the top ten species blasting against the NT (Nucleotide Sequence Database) from the NCBI was showed in Table 1, demonstrating that there was no obvious exogenous contamination during the library construction.

**Table 1:** The top ten species blasted against the nucleotide sequence database (NT)

Species	Number of reads	Percentage (%)
*Cyprinus carpio*	854	8.54
*Larimichthys crocea*	288	2.88
*Oryzias latipes*	90	0.90
*Danio rerio*	85	0.85
*Gouania willdenowi*	81	0.81
*Echeneis naucrates*	78	0.78
*Denticeps clupeoides*	74	0.74
*Syngnathus acus*	66	0.66
*Mastacembelus armatus*	65	0.65
*Salmo trutta*	59	0.59

### Genomic characteristics by K-mer analysis

About 364,763,910 clean reads were used to carry out *de novo* assembly based on K-mer analysis. Finally, a total length of 761,647,043 bp contigs were obtained with the contig N50 value of 1366 bp and N90 value of 469 bp, and the maximum contig was 18,910 bp in length. A 350-bp insert library data were used to construct the K-mer distribution map of *K* = 17 and the 17-mer frequency distribution curve exhibited a unique peak at depth of 24 (Figure 1B). Statistical analysis showed that the total number of K-mers was 47,338,914,261 after removing the anomalies of depth. According to the calculation formula of genome size, we counted the revised genome size of the diploid species *O. evermanni* was 1.97 Gb after eliminating the effects of erroneous K-mers. The proportion of heterozygosity and repeat sequence ratio were 0.70% and 43.30%, respectively.

### Gene prediction and annotation

A total of 9016 putative coding genes with the average length of 710 bp were successfully predicted by GeneMark-ES software (Table 2). The total length of genes and intergenic regions were 6,405,663 and 755,241,380 bp, with the GC content of 55.0% and 42.5%, respectively. The predicted genes were separately aligned by BLAST 2.2.28+ to the GO, KEGG, NR and KOG databases.

**Table 2:** Statistical information of predicted genes

Gene number	Gene total length (bp)	Gene average length (bp)	Gene density (genes/kb)	Intergenic region length (bp)	GC content in gene region (%)	GC content in intergenic region (%)
9,016	6,405,663	710	0.011	755,241,380	55.00	42.50

**Figure 2:**
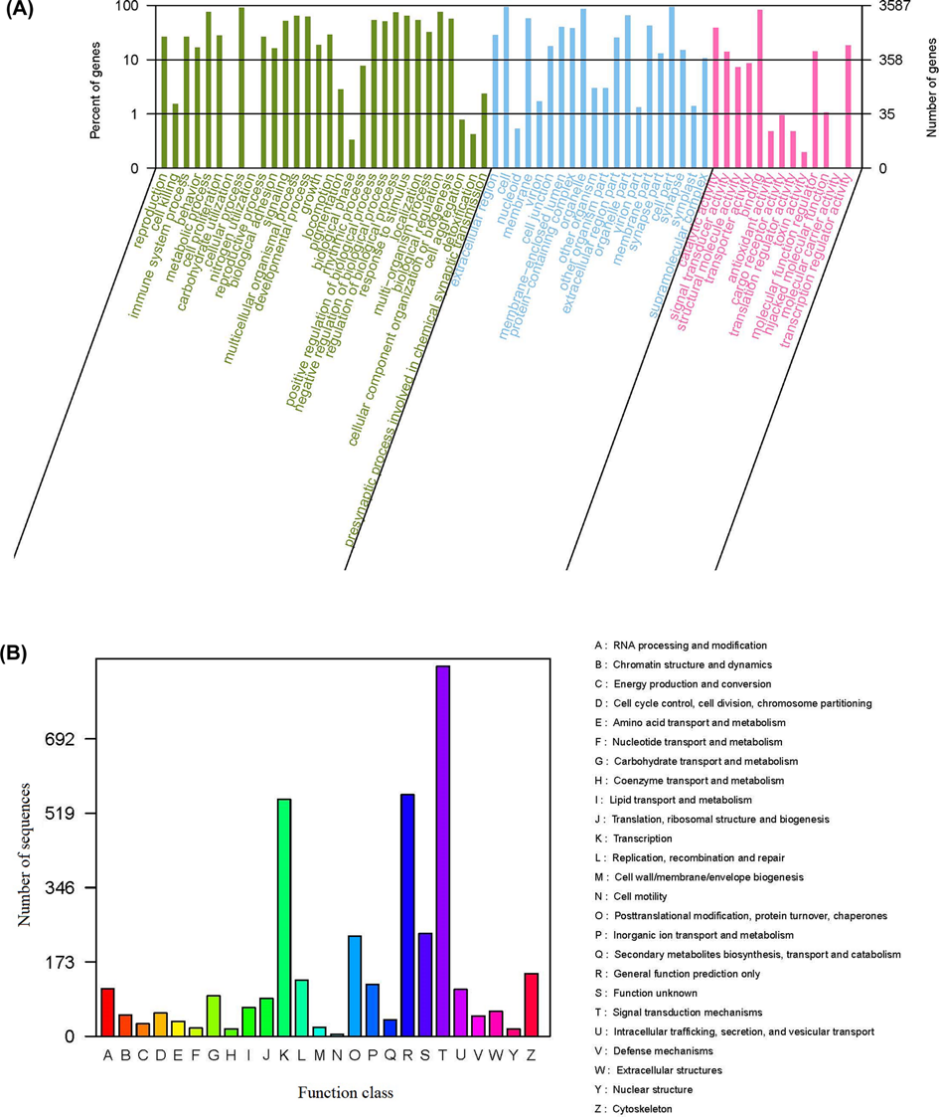
GO annotation and KOG function classification of putative genes in *O. evermanni* (**A**) Genes were assigned to three categories: biological process, cellular component and molecular function. (**B**) Different color codes (A–Z) at right of the histogram represented different category.

A total of 3587 unigenes were identified by GO analysis and further classified into the categories of molecular function, cellular component and biological process (Figure 2A). About 55.04% of them were grouped under biological processes, in which metabolic process was the most highly represented group. Second, 34.73% of the genes were grouped under cellular components, in which cell and cell part were the most significantly represented groups. Finally, 10.23% of the genes were grouped under molecular functions, in which binding represented a relatively high proportion. There were 4,375 genes were classified into KOG functional categories, the signal transduction mechanisms represented the largest group (861; 19.68%), followed by general function prediction only (562; 12.85%) and transcription (551; 12.59%) (Figure 2B).

Gene annotation analysis showed that a lot of predicted genes of *O. evermanni* genome were associated with the functional category of signal transduction mechanisms (861 genes) and immunity (950 genes). The functions of gene products in cells and their potential metabolic pathways were available in Supplementary Figure S2.

### Microsatellites distribution and characteristics

**Figure 3:**
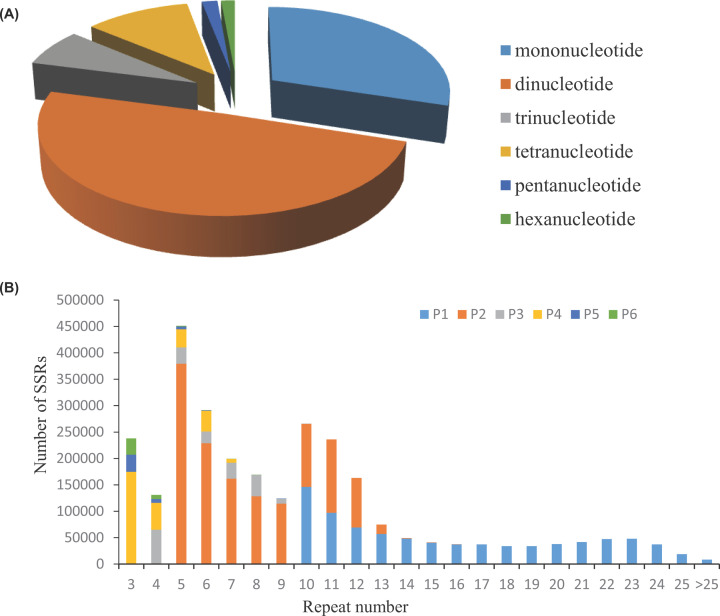
Distribution of SSR motifs in *O. evermanni* (**A**) Frequency of different microsatellite motif types. (**B**) Distributions of different motif types with different repeat numbers.

Microsatellite identification tool (MISA) was used for microsatellite mining. As a result, 9,382,261 sequences with a total length of 1,214,882,177 bp were examined, and 2,812,813 SSRs were finally identified. Totally 2,187,607 SSR-containing sequences were detected accounting for 23.32% the total examined sequences. Among them, about 485,719 sequences contained more than 1 SSR and the number of SSRs present in compound formation was 425,676. The most abundant type of repeat was the dinucleotide (1,383,575; 49.19%), followed by mononucleotide (839,597; 29.85%), tetranucleotide (306,611; 10.90%), trinucleotide (197,737; 7.03%), pentanucleotide (45,529; 1.62%) and hexanucleotide (39,764; 1.41%) repeats (Figure 3A). The most and the second most common repeat types were five times repeats (451,077) and six times repeats (291,123) (Figure 3B).

**Figure 4:**
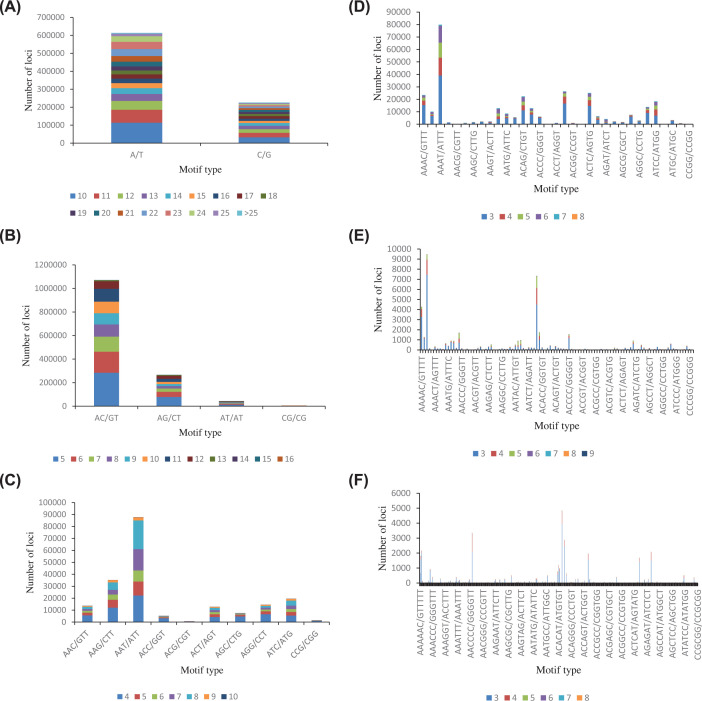
Type and frequency of microsatellite motifs in *O. evermanni* (**A**) Frequency of different mononucleotide microsatellite motifs. (**B**) Frequency of different dinucleotide microsatellite motifs. (**C**) Frequency of different trinucleotide microsatellite motifs. (**D**) Frequency of different tetranucleotide microsatellite motifs. (**E**) Frequency of different pentanucleotide microsatellite motifs. (**F**) Frequency of different hexanucleotide microsatellite motifs.

In this study, the dominant repeating motifs ranging from mononucleotide to hexanucleotide were A (363,365), CA (373,131), AAT (22,229), AAAT (24,328), AAAAT (2978) and CACACG (1227) of the total SSRs (Table 3). Among the dinucleotide motifs, the most abundant repeat motif type was AC/GT, followed by AG/CT, AT/AT and CG/CG. Within the trinucleotide repeat motifs, the major repeat motifs were AAT/ATT and AAG/CTT, accounting for 44.35% and 17.77%, respectively. Percentages of different motifs in mon-, tetra-, penta- and hexa- nucleotide repeats were also showed in Figure 4.

**Table 3:** Dominant base classes in each base repeat type in *O. evermanni*

Repeat type	Maximum repeat modify				Minimum repeat modify		
	Type	Repeat motif	Number	Proportion	Repeat motif	Number	Proportion
Mononucleotide	4	A	363,365	43.28%	G	66,406	7.91%
Dinucleotide	12	CA	373,131	26.97%	GC	2194	0.16%
Trinucleotide	60	AAT	22,229	11.24%	CGT	39	0.02%
Tetranucleotide	240	AAAT	24,328	7.93%	TCGT	10	0.003%
Pentanucleotide	966	AAAAT	2978	6.54%	–	–	–
Hexanucleotide	2361	CACACG	1227	3.09%	–	–	–

### Mitochondrial DNA structure and phylogenetic relationships

**Figure 5:**
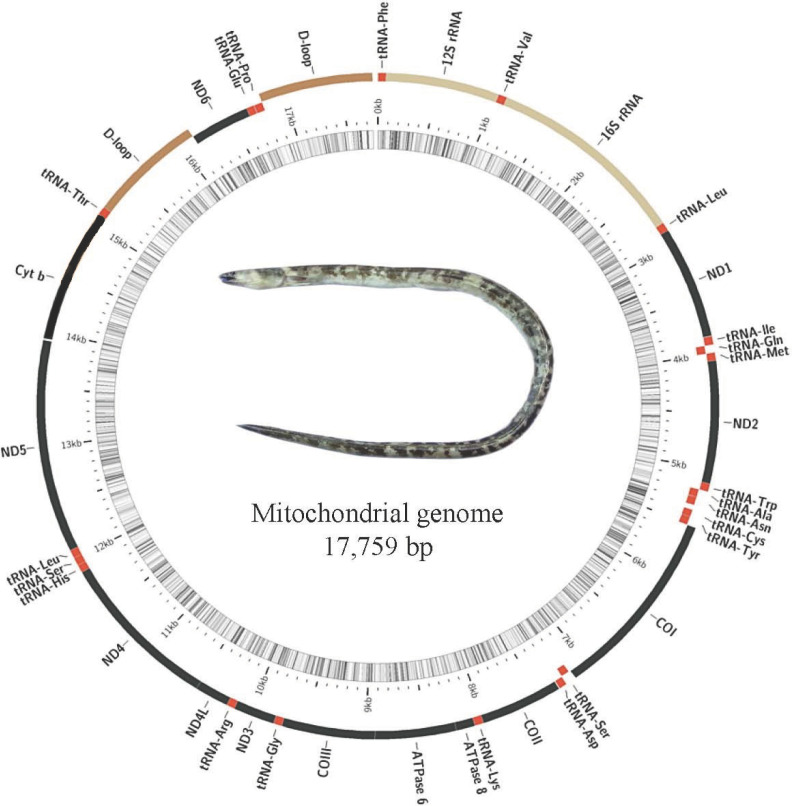
The mitochondrial genome structure of *O. evermanni*

It was the first time to report the complete mitogenome for *O. evermanni* in this study. The complete mitochondrial genome was 17,759 bp in length (Figure 5), with the base composition of A (31.27%), G (16.19%), C (26.22%) and T (26.32%), respectively. The A+T content (57.59%) was greater than G+C content (42.41%), showing an obvious AT bias. Unlike other typical teleosts, the gene arrangement was identified in the mitogenome of *O. evermanni*. ND6 gene and the conjoint tRNA-Glu were translocated between tRNA-Thr and tRNA-Pro, and another highly homologous D-loop region was located in the upstream of the ND6 gene. The tRNA-Gln (Q), tRNA-Ala (A), tRNA-Asn (N), tRNA-Cys (C), tRNA-Tyr (Y), tRNA-Ser^UCA^ (S1), tRNA-Glu (E), tRNA-Pro (P) and ND6 were located in the L-strand, while the rests were located in the H-strand. Except for tRNA-Ser (AGC), the remaining 21 tRNAs could fold into typical cloverleaf secondary structure.

**Figure 6:**
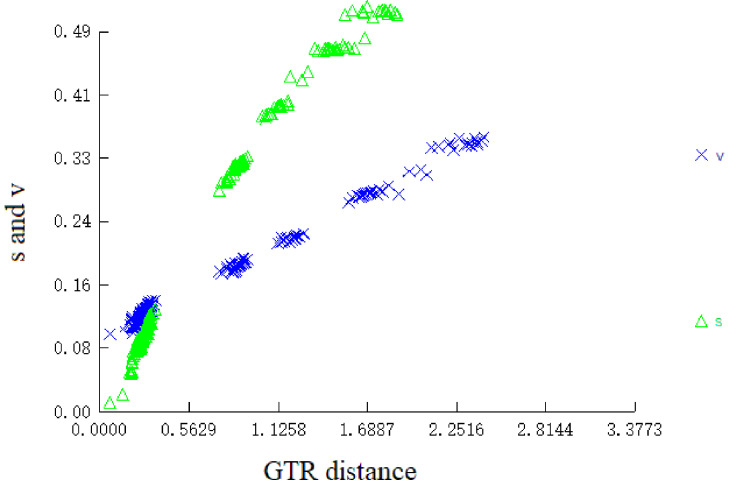
Nucleotide substitution saturation analysis of 12 PCGs sequences without ending codons

**Figure 7:**
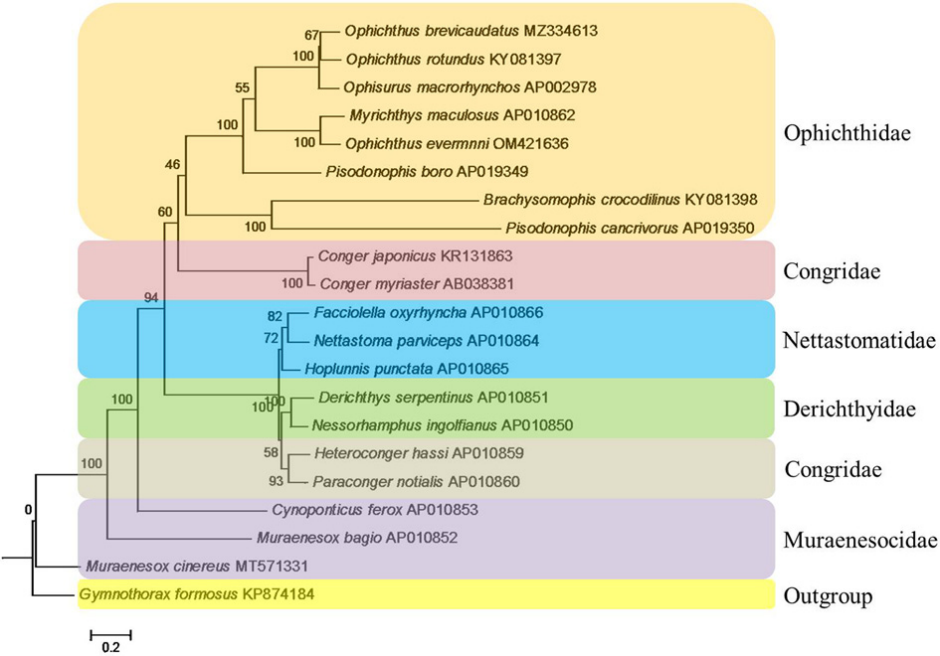
The phylogenetic tree inferred from the mitogenome sequences of 21 Anguilliformes fishes. Sample from this study was written in red letters

Phylogenetic relationships were constructed based on the linked sequences of 12 PCGs (without stop codons) of 21 mitogenomes using BI method. In order to make sure that the aligned sequences were suitable for tree construction, we conducted the test of substitution saturation based on *I*_ss_ statistic for the dataset with DAMBE prior to phylogenetic analysis. The observed *I*_ss_ value (0.3013) was significantly smaller than *I*_ss.c_ value (0.8496 assuming a symmetrical topology and 0.6444 assuming an extreme asymmetrical topology) when all three codon positions were considered as a whole. Furthermore, the plot trend-line analysis was carried out using generalized time reversible (GTR) distance as abscissa and base substitution as ordinate (Figure 6). The result showed that there was an obvious linear relationship between them, indicating the sequences obviously had experienced little substitution saturation and subsequent phylogenetic analysis was feasible. GTR+G model was chosen as the appropriate model for the nucleotide sequences based on Akaike information criterion (AIC). The reconstructed BI tree was showed in Figure 7. It revealed that all Ophichthidae species gathered as one clade, and *O. evermanni* had the closest relationship with *Myrichthys maculosus*. Family Ophichthidae clustered with one group of Congridae consisting of *Conger japonicus* and *C. myriaster*. Nettastomatidae, Derichthyidae and Congridae (*Heteroconger hassi* + *Paraconger notialis*) formed another clade. While, species of Muraenesocidae located near the root of the phylogenetic tree.

## Discussion

The family Ophichthidae, the most divergent group within the order Anguilliformes, comprises two subfamilies, the Myrophinae and the Ophichthinae, the latter of which is characterized by a hard, pointed and finless tail tip [3,4]. The genus *Ophichthus* includes the highest numbers of species (285 valid species worldwide) among all of the 47 recognized genera in the subfamily Ophichthinae, and there have been some new species to be constantly discovered and reported since the last two decades [1]. Therefore, the classification and identification of the snake eels are always in the utmost confusion [34]. Fourteen-four species of *Ophichthus* were recorded and described in offshore China, which mainly distributed in the sea waters south of the Yangtze River Estuary [2]. However, very limited molecular genetic researches have been focused on a certain snake eel both at home and abroad.

In the present study, the Illumina paired-end sequencing technique was applied to preliminarily unravel the genomic background of *O. evermanni*, a representative species of this group. Genome size refers to the amount of DNA contained in a haploid genome, and it serves as an important basis for comparative and evolutionary genomics [35]. Genome size and its variability were influenced by the mutational pressure of chromosomal, transposon activity and relative occurrence rates of segmental duplications and deletions to some extent [36]. The draft genome size of *O. evermanni* was relatively larger than those of most marine teleosts, such as *Fugu rubripes* (322.5 Mb) [10], *Gadus morhua* (830 Mb) [37], *Larimichthys crocea* (728 Mb) [13], *Sillago sinica* (534 Mb) and [38]. Previous researches indicated larger genomes had relatively higher mutational liability to undergoing natural selection in evolutionary process [39], and lungfish was a good case in point [40]. Our result implied that larger genomes of snake eels might accumulate more mutations and have strong ability to adapting to the benthic and burrowing living habits in sandy shores or muddy estuaries.

Genome size is determined not only by the number of genes in the genome but also by the amount of repetitive DNA. The repeat ratio (43.30%) of *O. evermanni* genome was present at a high level in the known fish genomes. It confirmed that larger genomes tended to be ones in which the copy numbers of the repeat sequences were highest [41]. Heterozygosity is important for determining the appropriate genomic splicing strategy and subsequence data processing. The genome-scale de novo assembly will become difficult when the heterozygosity exceeds 0.5% [22]. According to the criteria, the higher proportion of heterozygosity (0.70%) reflected the complexity of *O. evermanni* genome, and also inferred higher genetic diversity in *O. evermanni.* Low (<25%) or high (>65%) GC content may cause sequencing bias of Illumina platform and seriously affect the quality of genome assembly and subsequent analysis [42]. In the study, the moderate GC content was detected and the percentage of A vs. G and C vs. T were almost equal to each other, indicating the sequencing quality was good and suitable for further analysis.

As cave-dwelling fish species, the visual system structure and function of the snake eels have degenerated dramatically, by contrast, the olfactory organs and lateral line canals are well developed [43,44]. In the present study, some signal transduction pathways (MAPK signaling pathway, olfactory transduction, taste transduction, neuroactive ligand–receptor interaction etc.) were detected and therefore environmental messages are received from the sensory organs and then abundant nerve fibers can transmit external stimulus to the brain. In addition, some signaling pathways related to immune system were also founded, such as intestinal immune network for B-cell receptor signaling pathway, T-cell receptor signaling pathway, Jak-STAT signaling pathway, NOD-like receptor signaling pathway and Toll-like receptor signaling pathway. In coastal areas of Guangdong and Fujian, China, local residents regard it as healthy tonic for strengthening body and improving immunity.

Microsatellite DNA marker offers several advantages of codominant, extensive distribution, abundant polymorphisms and convenient analysis, and has become an ideal tool in genetics and evolution studies [45]. In the present study, the dinucleotide repeats had the highest number and type of repeats, which was consistent with *Acanthogobius ommaturus* [46], *Sillago sihama* [47], *Harpadon nehereus* [48] and *Cociella crocodilus* [49]. SSR polymorphic loci are mainly distributed in dinucleotide and trinucleotide repeats [50]. Hence, the development of polymorphic SSR markers from low repetitive motifs will have great potential in population genetics research of *O. evermanni* subsequently. The complexity of repeated motif is usually related to evolutionary level and DNA mutation rate [51]. The frequency of mononucleotides to trinucleotides was amount to 86.07%, which implied that *O. evermanni* might have experienced a long evolutionary history and accumulated more genetic variation. Apart from SSRs, another important molecular marker mitochondrial DNA was also assembled to explore the systematical evolution of *O. evermanni.* The intricate clustering relationship in family Ophichthidae was presented in the topological structure of BI tree, deducing that Ophichthidae was not a monophyletic group and should be a polyphyletic group. The conclusion was identical with morphological and anatomical evidences of olfactory organs [44]. The snake eels have later divergence time on evolution comparing to other related species, and the short interval time of differentiation might cause a rapid affair of evolution radiation and species forming in this group.

## Conclusions

In the present study, the genome size of *O. evermann* estimated by K-mer analysis (*K* = 17) was 1.97 Gb, with the heterozygosity and duplication rates 0.70% and 43.30%, respectively. The results showed *O. evermann* owned relatively larger genome size, higher heterozygosity and nucleotide repetition ratio in bony fishes. Besides, the gene annotation, SSR characteristics and phylogenetic relationship analyses were tentatively carried out. Our results would provide meaningful data for further genomic studies and lay a useful basis for novel molecular marker development. Because genome size based on K-mer analysis might be affected by data quality, analytical software, parameters setting and some other confounding factors. Hence, the novel state-of-the-art genetic techniques, such as Illumina combined with PacBio and Hi-C-based assembly needs to be conducted to obtain chromosomal-level scaffolding genome in the future.

## Supplementary Material

Supplementary Figures S1-S2

## Data Availability

The data presented in this study are openly available in NCBI database.

## Abbreviations

AIC: Akaike information criterion
BI: Bayesian inference
GO: gene ontology
GSS: genome survey sequencing
GTR: generalized time reversible
MCMC: Markov Chain Monte Carlo
MISA: microsatellite identification tool
NGS: next-generation sequencing
PCG: protein-coding gene
