# Supplementary material for: Genome-wide survey and genetic characteristics of Ophichthus evermanni based on Illumina sequencing platform
Source: Biosci Rep. 2022 May 27;42(5):BSR20220460. doi: 10.1042/BSR20220460 (PMC9142832; doi:10.1042/BSR20220460)
Supplement: Supplementary Figures S1-S2 [file BSR-2022-0460_supp.pdf]

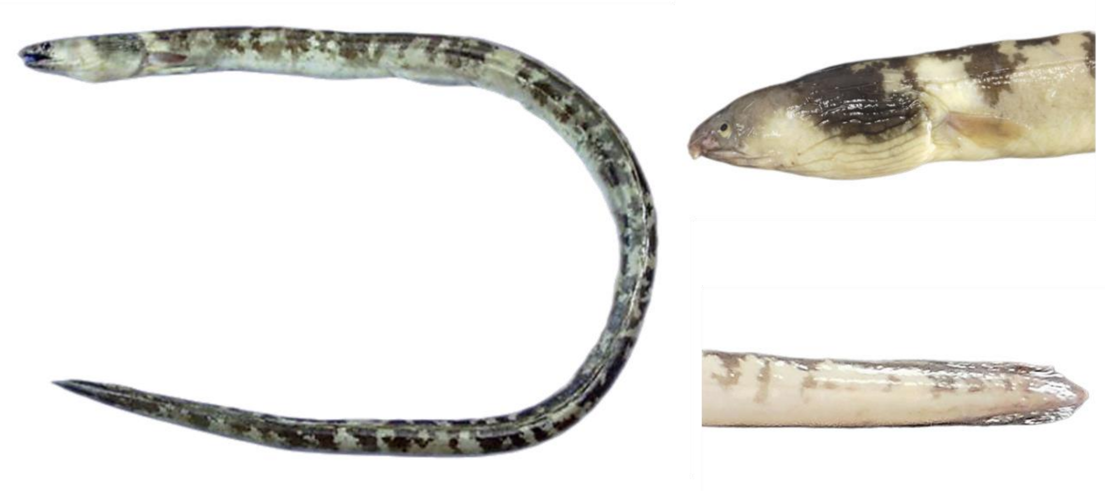

Supplementary Figure 1. The morphological picture of *O. evermanni*.

(A)

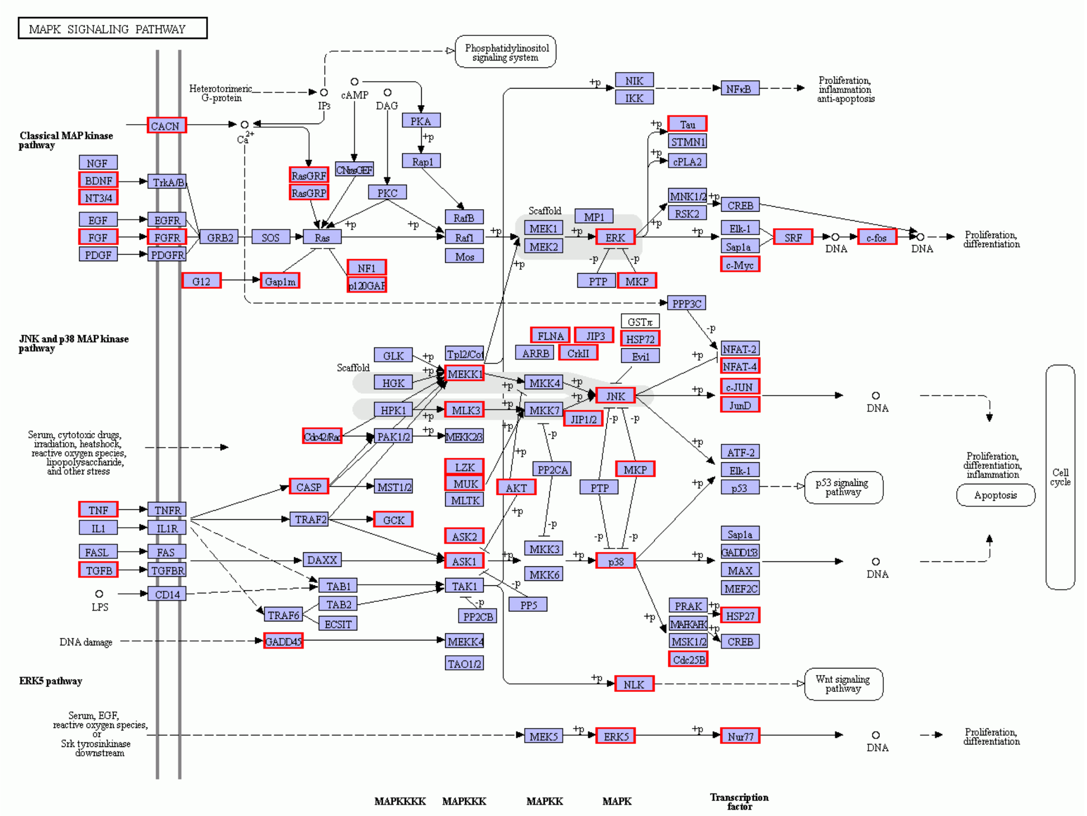

(B)

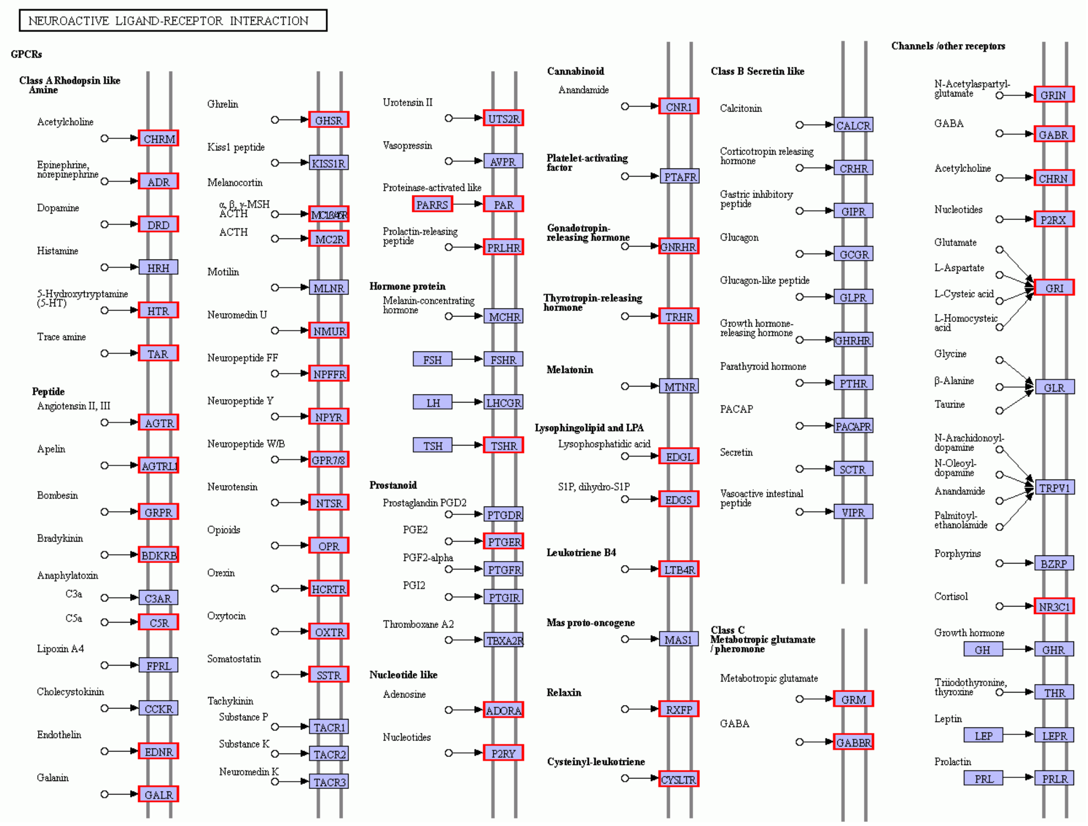
NEUROACTIVE LIGAND-RECEPTOR INTERACTION
GPCRs
Class A Rhodopsin like
Amine
Acetylcholine
CHRM
Epinephrine, norepinephrine
ADR
Dopamine
DRD
Histamine
HRH
5-Hydroxytryptamine (5-HT)
HTR
Trace amine
TAR
Peptide
Angiotensin II, III
AGTR
Apelin
AGTRL1
Bombesin
GRPR
Bradykinin
BDKRB
Anaphylatoxin
C3a
C3AR
C5a
C5R
Lipoxin A4
FPRL
Cholecystokinin
CCKR
Endothelin
EDNR
Galanin
GALR
Ghrelin
GHSR
Kiss1 peptide
KISS1R
Melanocortin
α, β, γ-MSH
ACTH
ACTH
MC2R
Motilin
MLNR
Neuromedin U
NMUR
Neuropeptide FF
NPFFR
Neuropeptide Y
NPYR
Neuropeptide W/B
GPR7/8
Neurotensin
NTSR
Opioids
OPR
Orexin
HCRTR
Oxytocin
OXTR
Somatostatin
SSTR
Tachykinin
Substance P
TACR1
Substance K
TACR2
Neuromedin K
TACR3
Urotensin II
UTS2R
Vasopressin
AVPR
Proteinase-activated like
PARRS
PAR
Prolactin-releasing peptide
PRLHR
Hormone protein
Melanin-concentrating hormone
MCHR
FSH
FSHR
LH
LHCGR
TSH
TSHR
Prostanoid
Prostaglandin PGD2
PTGDR
PGE2
PTGER
PGF2-alpha
PTGFR
PGI2
PTGIR
Thromboxane A2
TBXA2R
Nucleotide like
Adenosine
ADORA
Nucleotides
P2RY
Cannabinoid
Anandamide
CNR1
Platelet-activating factor
PTAFR
Gonadotropin-releasing hormone
GNRHR
Thyrotropin-releasing hormone
TRHR
Melatonin
MTNR
Lysophospholipid and LPA
Lysophosphatidic acid
EDGL
S1P, dihydro-S1P
EDGS
Leukotriene B4
LTB4R
Mas proto-oncogene
MAS1
Relaxin
RXFP
Cysteinyl-leukotriene
CYSLTR
Class B Secretin like
Calcitonin
CALCR
Corticotropin releasing hormone
CRHR
Gastric inhibitory peptide
GIPR
Glucagon
GCGR
Glucagon-like peptide
GLPR
Growth hormone-releasing hormone
GHRHR
Parathyroid hormone
PTHR
PACAP
PACAPR
Secretin
SCTR
Vasoactive intestinal peptide
VIPR
Class C Metabotropic glutamate / pheromone
Metabotropic glutamate
GRM
GABA
GABBR
Channels /other receptors
N-Acetylaspartyl-glutamate
GRIN
GABA
GABR
Acetylcholine
CHRN
Nucleotides
P2RX
Glutamate
L-Aspartate
L-Cysteic acid
L-Homocysteic acid
GRI
Glycine
β-Alanine
Taurine
GLR
N-Arachidonoyl-dopamine
N-Oleoyl-dopamine
Anandamide
Palmitoyl-ethanolamide
TRPV1
Porphyrins
BZRP
Cortisol
NR3C1
Growth hormone
GH
GHR
Triiodothyronine, thyroxine
THR
Leptin
LEP
LEPR
Prolactin
PRL
PRLR

(C)

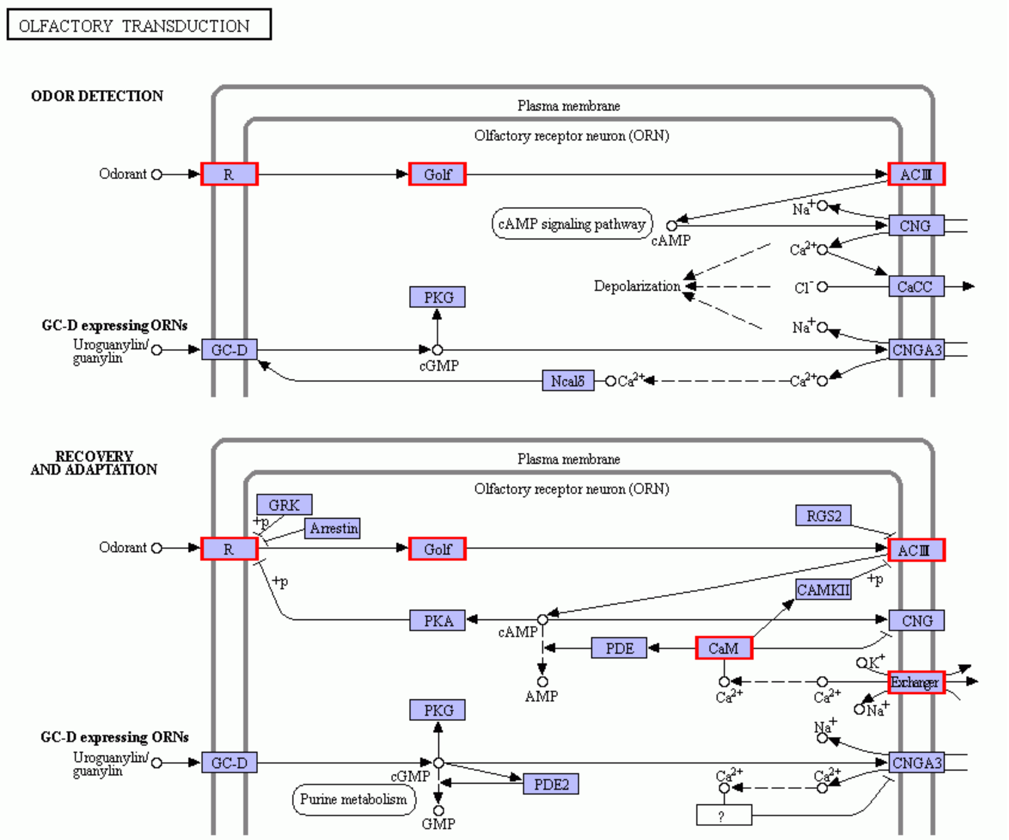
OLFACTORY TRANSDUCTION
ODOR DETECTION
Plasma membrane
Olfactory receptor neuron (ORN)
Odorant
R
Golf
ACIII
cAMP signaling pathway
cAMP
Na+
CNG
Ca2+
Depolarization
Cl-
CaCC
PKG
GC-D expressing ORNs
Uroguanylin/ guanylin
GC-D
cGMP
Na+
CNGA3
Ncalδ
Ca2+
Ca2+
RECOVERY AND ADAPTATION
Plasma membrane
Olfactory receptor neuron (ORN)
GRK
+p
Arrestin
Odorant
R
Golf
RGS2
ACIII
+p
CAMKII
+p
PKA
cAMP
CNG
PDE
CaM
AMP
K+
Exchanger
Ca2+
Ca2+
Na+
PKG
Na+
GC-D expressing ORNs
Uroguanylin/ guanylin
GC-D
cGMP
PDE2
CNGA3
Purine metabolism
GMP
Ca2+
Ca2+
?

(D)

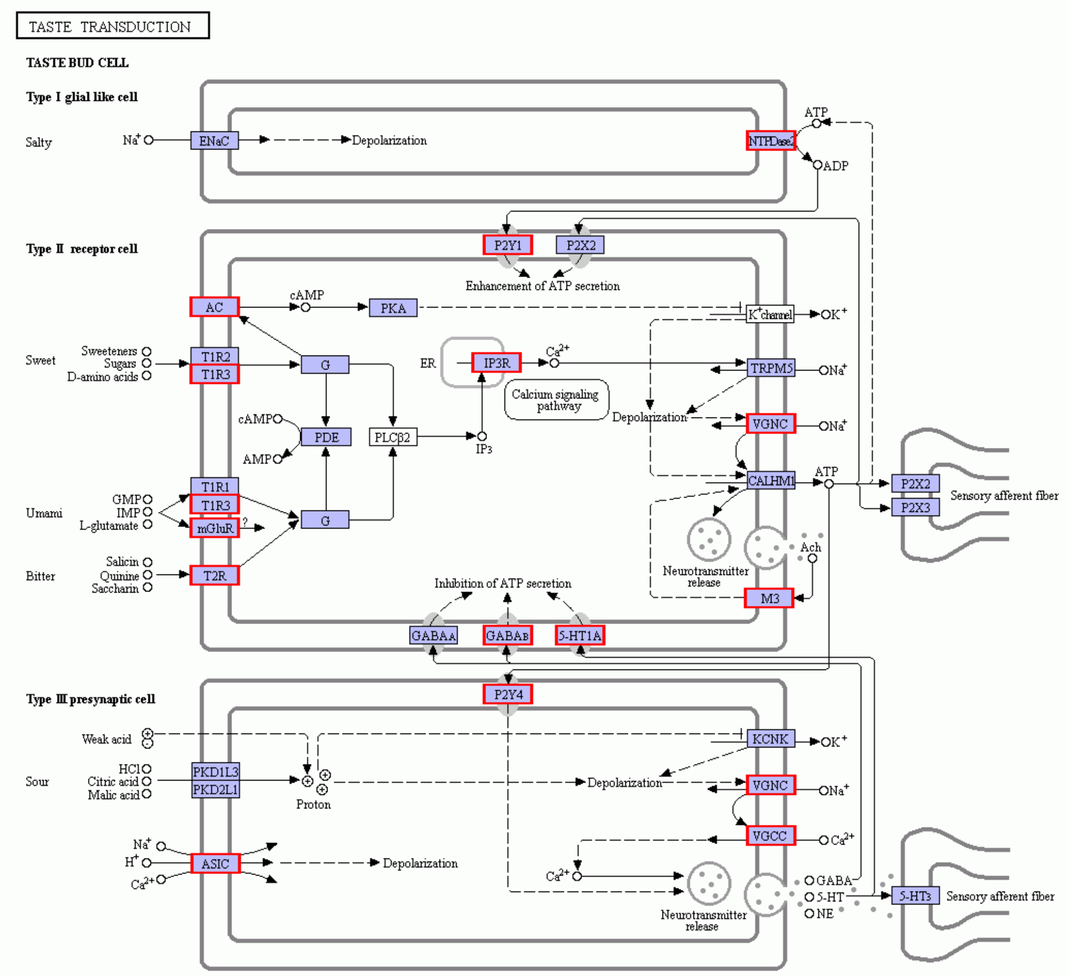
TASTE TRANSDUCTION
TASTE BUD CELL
Type I glial like cell
Type II receptor cell
Type III presynaptic cell
Salty
Sweet
Umami
Bitter
Sour
ENaC
Depolarization
NTPDase2
ATP
ADP
P2Y1
P2X2
Enhancement of ATP secretion
AC
cAMP
PKA
K+ channel
T1R2
T1R3
G
ER
IP3R
Ca2+
TRPM5
Calcium signaling pathway
PDE
PLCβ2
IP3
VGNC
CALHM1
T1R1
mGluR
T2R
P2X3
Sensory afferent fiber
Neurotransmitter release
Ach
M3
Inhibition of ATP secretion
GABAA
GABAB
5-HT1A
P2Y4
Weak acid
KCNK
PKD1L3
PKD2L1
Proton
VGCC
ASIC
GABA
5-HT
NE
5-HT3
Sweeteners
Sugars
D-amino acids
GMP
IMP
L-glutamate
Salicin
Quinine
Saccharin
HCl
Citric acid
Malic acid

(E)

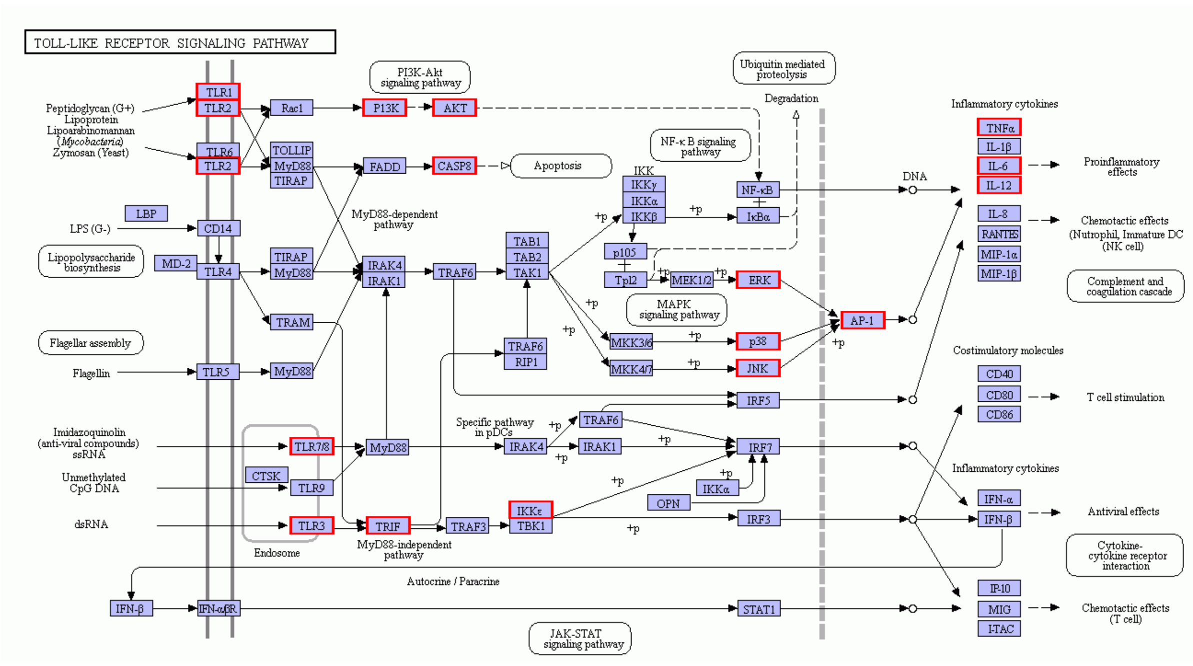
TOLL-LIKE RECEPTOR SIGNALING PATHWAY
Peptidoglycan (G+)
Lipoprotein
Lipoarabinomannan
(Mycobacteria)
Zymosan (Yeast)
TLR1
TLR2
TLR6
Rac1
PI3K
AKT
PI3K-Akt signaling pathway
Ubiquitin mediated proteolysis
Degradation
TOLLIP
MyD88
TIRAP
FADD
CASP8
Apoptosis
NF-κB signaling pathway
LBP
LPS (G-)
CD14
Lipopolysaccharide biosynthesis
MD-2
TLR4
IRAK4
IRAK1
TRAF6
TAB1
TAB2
TAK1
IKK
IKKγ
IKKα
IKKβ
NF-κB
IκBα
p105
Tpl2
MEK1/2
ERK
MAPK signaling pathway
MyD88-dependent pathway
TRAM
Flagellar assembly
Flagellin
TLR5
RIP1
MKK3/6
MKK4/7
p38
JNK
AP-1
IRF5
DNA
Inflammatory cytokines
TNFα
IL-1β
IL-6
IL-12
Proinflammatory effects
IL-8
RANTES
MIP-1α
MIP-1β
Chemotactic effects
(Nutrophil, Immature DC
(NK cell)
Complement and coagulation cascade
Costimulatory molecules
CD40
CD80
CD86
T cell stimulation
Imidazoquinolin
(anti-viral compounds)
ssRNA
Unmethylated CpG DNA
dsRNA
TLR7/8
CTSK
TLR9
TLR3
Endosome
Specific pathway in pDCs
IRAK4
IRAK1
IRF7
IKKα
OPN
TRIF
TRAF3
IKKε
TBK1
IRF3
MyD88-independent pathway
IFN-α
IFN-β
Antiviral effects
Cytokine-cytokine receptor interaction
Autocrine / Paracrine
IFN-β
IFN-α/βR
STAT1
JAK-STAT signaling pathway
IP-10
MIG
I-TAC
Chemotactic effects
(T cell)
+p

(F)

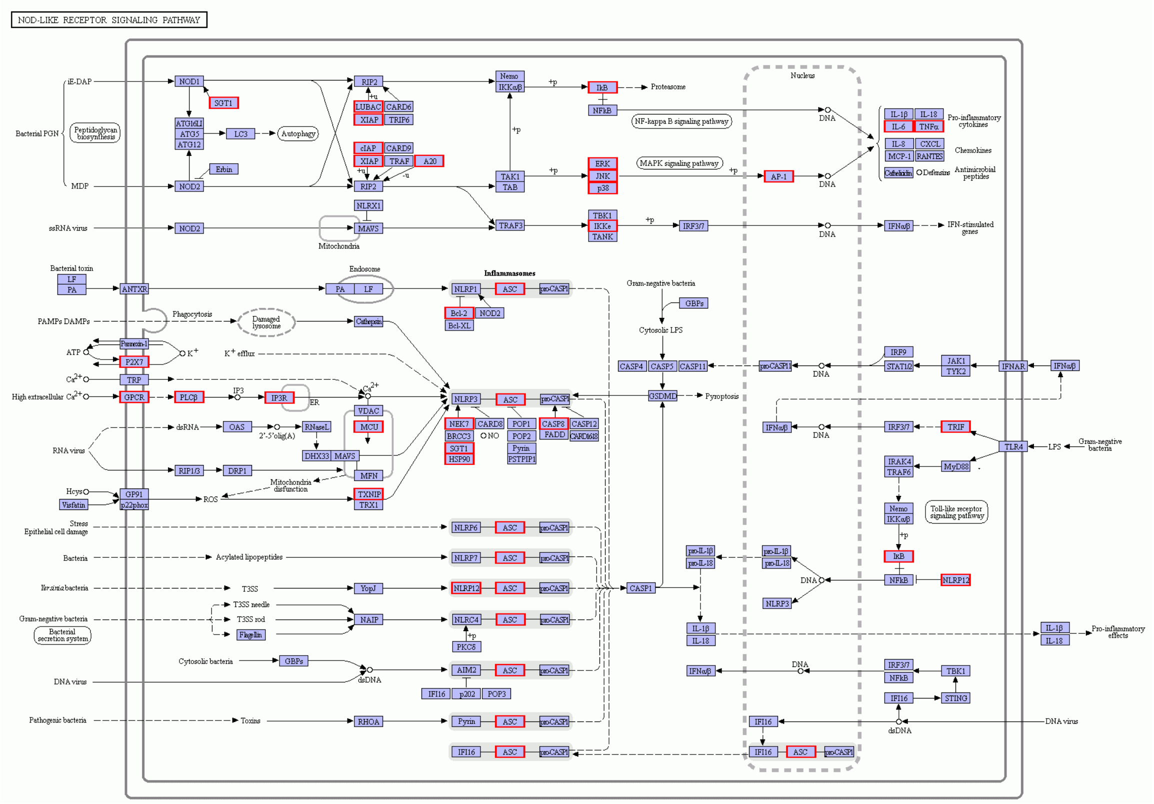
NOD-LIKE RECEPTOR SIGNALING PATHWAY

(G)

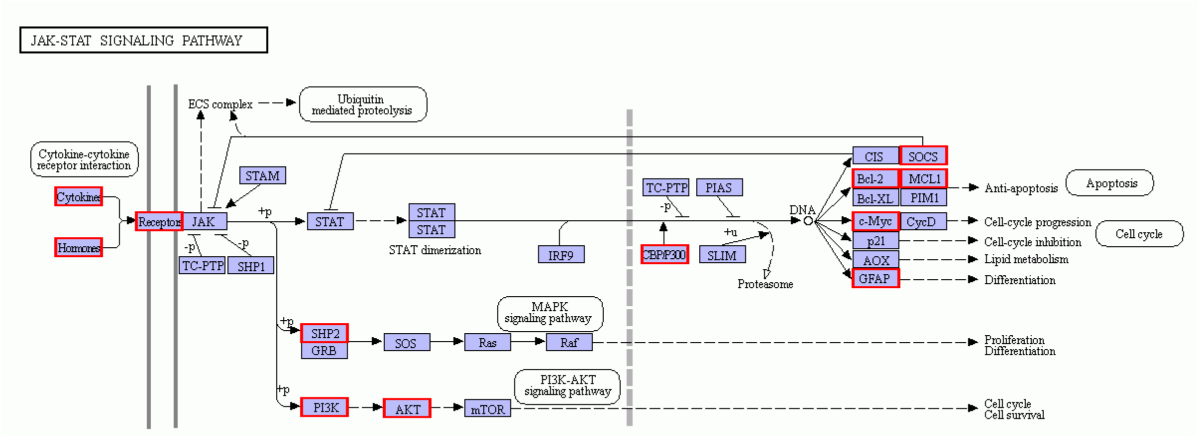
JAK-STAT SIGNALING PATHWAY
Cytokine-cytokine receptor interaction
Cytokines
Hormones
Receptor
JAK
ECS complex
Ubiquitin mediated proteolysis
STAM
STAT
TC-PTP
SHP1
STAT dimerization
IRF9
PIAS
CBP/P300
SLIM
Proteasome
DNA
CIS
SOCS
Bcl-2
MCL1
Bcl-XL
PIM1
c-Myc
CycD
p21
AOX
GFAP
Anti-apoptosis
Apoptosis
Cell-cycle progression
Cell-cycle inhibition
Cell cycle
Lipid metabolism
Differentiation
SHP2
GRB
SOS
Ras
Raf
MAPK signaling pathway
Proliferation Differentiation
PI3K
AKT
mTOR
PI3K-AKT signaling pathway
Cell cycle Cell survival

(H)

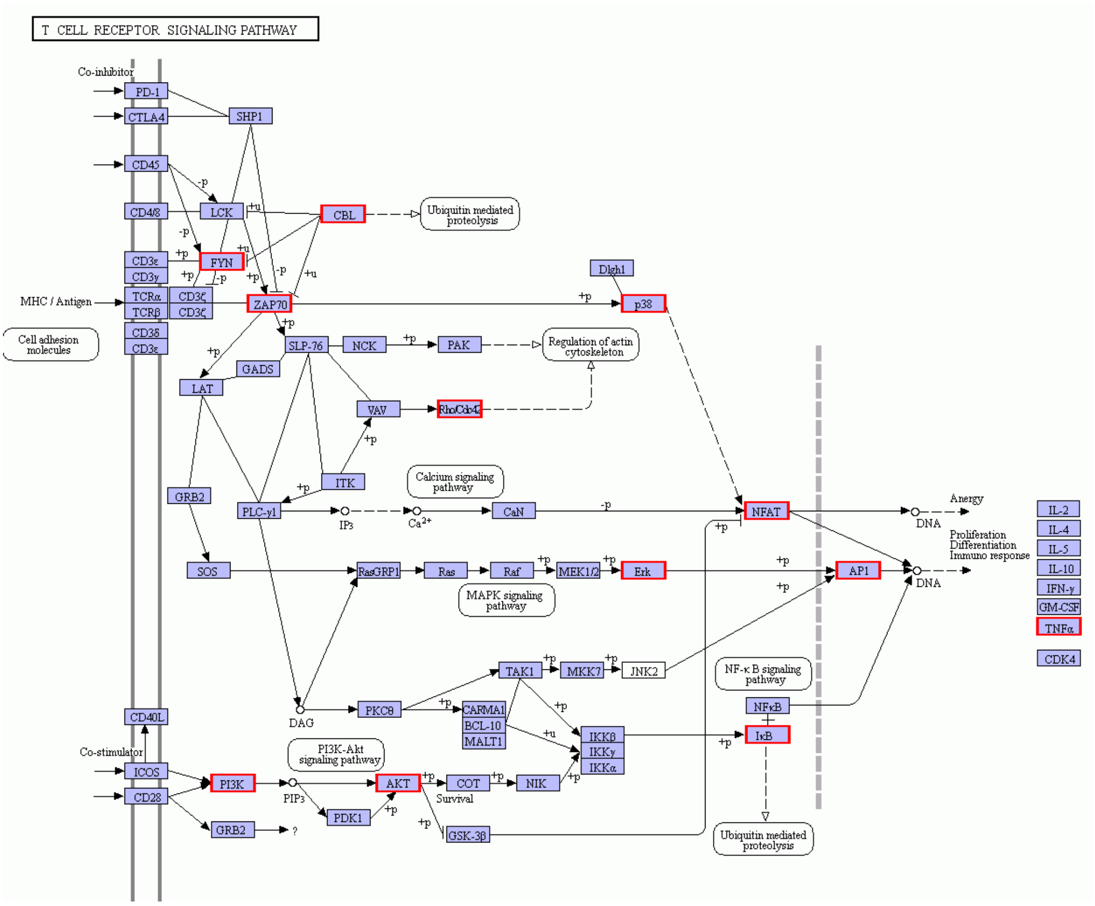

(I)

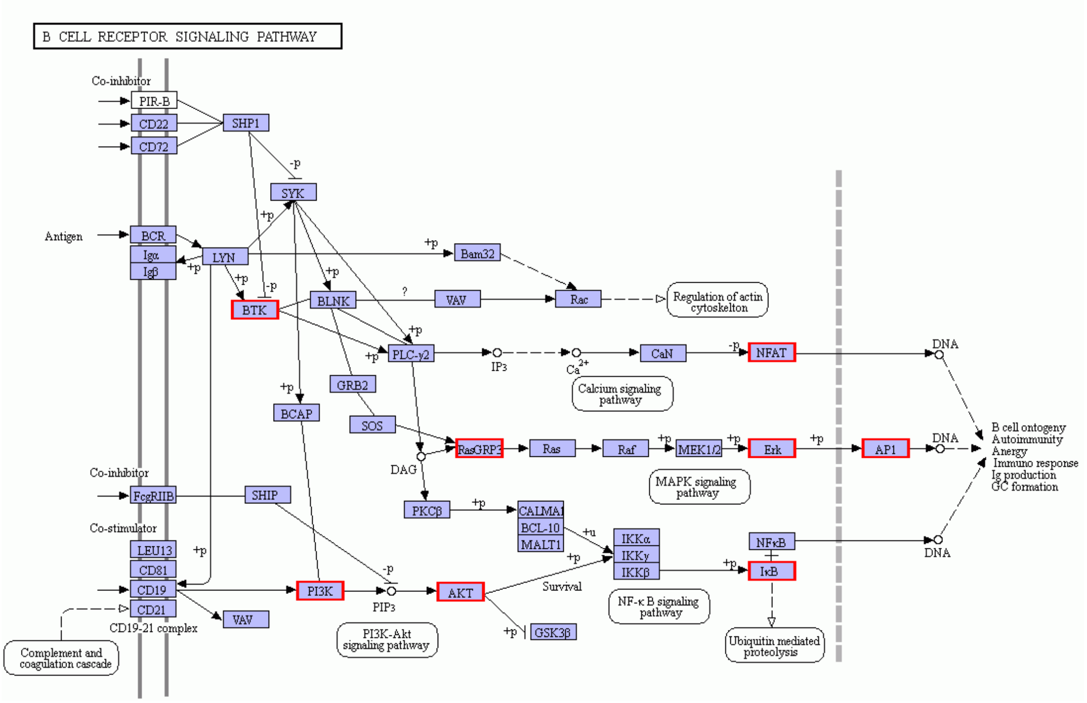

Supplementary Figure 2. The potential pathways involved in signal transduction mechanisms and immunity. (A) MAPK signaling pathway. (B) Neuroactive ligand-receptor interaction. (C)

Olfactory transduction. (D) Taste transduction. (E) Toll-like receptor signaling pathway. (F) NOD-like receptor signaling pathway. (G) Jak-STAT signaling pathway. (H) T cell receptor signaling pathway. (I) B cell receptor signaling pathway.
